# Machine Learning Algorithm-Based Prediction of Diabetes Among Female Population Using PIMA Dataset

**DOI:** 10.3390/healthcare13010037

**Published:** 2024-12-29

**Authors:** Afshan Ahmed, Jalaluddin Khan, Mohd Arsalan, Kahksha Ahmed, Abdelaaty A. Shahat, Abdulsalam Alhalmi, Sameena Naaz

**Affiliations:** 1 Microbial & Pharmaceutical Biotechnology Laboratory, Department of Pharmacognosy & Phytochemistry, School of Pharmaceutical Education and Research, Jamia Hamdard, Delhi 110062, India; afshanpari75@gmail.com (A.A.); jalaluddinkhan@jamiahamdard.ac.in (J.K.); 2 Department of Computer Science and Engineering, St. Andrews Institute of Technology & Management (SAITM), Gurugram 122506, India; arsalan_cse@saitm.ac.in; 3 Department of Pharmacognosy, College of Pharmacy, King Saud University, P.O. Box 2457, Riyadh 11451, Saudi Arabia; ashahat@ksu.edu.sa; 4 Department of Pharmaceutics, School of Pharmaceutical Education and Research, Jamia Hamdard, Delhi 110062, India; asalamahmed5@gmail.com; 5 Department of Computer Science, School of Arts, Humanities and Social Sciences, University of Roehampton, London SW15 5PH, UK; sameena.naaz@roehampton.ac.uk; 6 Department of Computer Science & Engineering, School of Engineering Sciences & Technology, Jamia Hamdard, Delhi 110062, India

**Keywords:** machine learning, diabetes, random forest, decision tree, Naïve Bayes, logistic regression

## Abstract

**Background**: Diabetes is a metabolic disorder characterized by increased blood sugar levels. Early detection of diabetes could help individuals to manage and delay the progression of this disorder effectively. Machine learning (ML) methods are important in forecasting the progression and diagnosis of different medical problems with better accuracy. Although they cannot substitute the work of physicians in the prediction and diagnosis of disease, they can be of great help in identifying hidden patterns based on the results and outcome of disease. **Methods**: In this research, we retrieved the PIMA dataset from the Kaggle repository, the retrieved dataset was further processed for applied PCA, heatmap, and scatter plot for exploratory data analysis (EDA), which helps to find out the relationship between various features in the dataset using visual representation. Four different ML algorithms Random Forest (RF), Decision Tree (DT), Naïve Bayes (NB), and Logistic regression (LR) were implemented on Rattle using Python for the prediction of diabetes among the female population. **Results**: Results of our study showed that RF performs better in terms of accuracy of 80%, precision of 82%, error rate of 20%, and sensitivity of 88% as compared to other developed models DT, NB, and LR. **Conclusions**: Diabetes is a common problem prevailing across the globe, ML-based prediction models can help in the prediction of diabetes much earlier before the worsening of the condition.

## 1. Introduction

Diabetes is a chronic disorder characterized by an abnormal increase in blood glucose levels. It is mainly categorized into two types, Type-1 and Type-2 diabetes. Type-1 diabetes is also known as insulin-dependent diabetes mellitus (IDDM), which cannot be managed by using oral hypoglycemic agents. Type-2 diabetes mellitus, also called non-insulin-dependent diabetes mellitus (NIDDM), can be managed by oral hypoglycemic agents [1].

The World Health Organization (WHO) defines diabetes as a global health problem, as per recent reports that 8.5% of the world population in 2014 was suffering from diabetes, wherein type-2 diabetes accounts for 95% of cases. Mortality among diabetes patients is also increasing day by day. In 2019, 1.5 million deaths were caused by diabetes, which accounted for 48% of all deaths. Data also state that between 2000 to 2019, there was a 3% increase in mortality from diabetes and it was 13% in lower-middle-income countries [2,3,4].

Diabetes is a metabolic disorder that cannot be cured completely; the patient has to take medicine lifelong to manage their increased blood glucose level and further reduce diabetes-related complications. Initially, it does not show any symptoms, but later on, the patient will feel thirsty and have frequent urination, blurred vision, tiredness, and reduced body weight. If it is not managed at an early stage, it can also damage blood vessels in the heart, eyes, kidneys, and nerves. Diabetes patients have higher chances of developing heart attacks, strokes, and kidney failure [5,6].

Although diabetes affects both male and female populations equally, women are at higher risk of developing diabetes-related complications [7] as compared to men, and the consequences of diabetes are more serious in women. It has been found that heart attack risk in female diabetic patients is six times more as compared to non-diabetic females, whereas, in males, it was two times more in diabetic males as compared to non-diabetic males; in addition, studies have shown that mortality in the diabetic female population is 16.9% higher as compared to non-diabetic females [8]. Another link between diabetes and related deaths among the female population shows that there is a 13% higher risk of death from all complications, a 30% higher chance of death due to cardiovascular complications, and a 58% higher risk of death due to coronary heart disease [9]. 

Traditionally, healthcare providers diagnose depending on the signs and symptoms of the disease; they do not focus on the future implications but rather provide treatment based on the current illness, which sometimes leads to the diagnosis of a disease. Nowadays, the use of electronic medical data is increasing day by day, which makes it possible to identify hidden patterns and forecast feature implications based on the history and current medical condition of the patient [10].

Early diagnosis of diabetes is crucial for effective management and to reduce the chances of diabetes-related complications [7]. ML methods are currently helpful in forecasting the development of various diseases including diabetes [11,12]. Considering the individual lifestyle, medical history, and family history, one can foresee the development of this metabolic disorder. It is also helpful for the healthcare provider to have an early diagnosis for effective and precise management of the disease [13].

**Figure 1:**
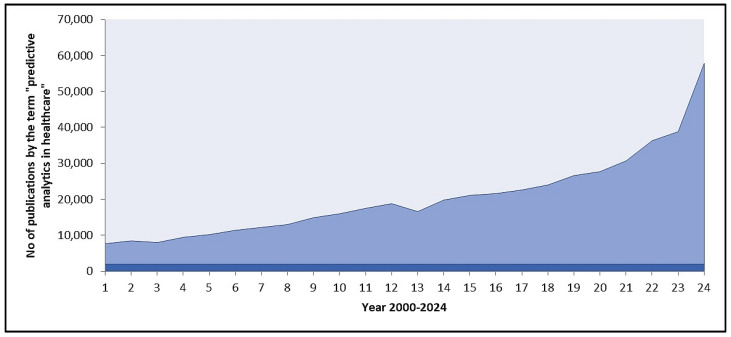
The chart depicts the increasing trend in the number of publications related to “predictive analytics in healthcare” from the year 2000 to 2024.

ML methods are computational methods that work based on datasets provided during model training. Nowadays, the method of diagnosis and treatment is changing, various computational tools are used to diagnose and predict the development of a disease using analysis of digital data that we are generating every day [14] ML is not only helpful in the diagnosis and prediction of disease but also for every aspect of drug development, from the discovery of a new molecule to the application of an existing molecule in a different medical application, known as drug repurposing [15,16,17]. Various ML algorithms have recently been used to develop artificial intelligence models to forecast diabetes [18]. Recent data show that the number of publications on the prediction of diabetes has been consistently increasing from 2000 to 2024 (Figure 1).

DT, RF, NB, and LR are the traditional methods used to predict diabetes. To reduce prediction errors and avoid misdiagnosis, we developed various ML models to predict diabetes in women using the PIMA dataset. These models aim to support healthcare providers in detecting diabetes early and preventing complications. The accuracy, precision, sensitivity, specificity, and F-score of the different ML models developed in this study were compared with each other and with results from earlier studies.

## 2. Materials and Methods

### 2.1. Dataset

In this work, the Pima Indian diabetes dataset (PID) was retrieved from the Kaggle ML Repository (https://www.kaggle.com, access on 19 October 2023) [19]. All the patients enrolled in this study were adult women who had reached the age of 21 and above. A brief overview of the dataset’s features is represented in Table 1, which includes data about 768 patients who took part in the clinical investigation, eight different parameters Pregnancies, blood glucose, blood pressure, skin thickness, insulin, body mass index (BMI) diabetes pedigree function (DPF), and age was taken, and the outcome was selected as the target.

**Figure 2:**
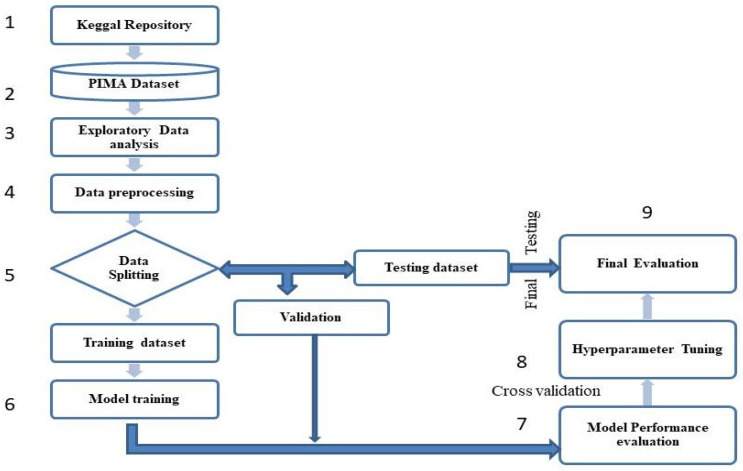
Flow chart of the methodology used for the ML model development.

The ‘outcome’ is the dependent or target variable, while the other eight attributes were independent variables. The ‘outcome’ variable for diabetes was a binary number, where 0 denotes no diabetic and 1 suggests diabetic. In this study, the accuracy of predicting a patient’s diabetes status in female patients was improved by utilizing data mining and ML algorithms. The methodology used in this work is depicted in Figure 2.

### 2.2. Exploratory Data Analysis (EDA)

Before developing the ML models, feature selection was conducted to identify important attributes in the dataset. To achieve this, correlation analysis was performed to explore relationships between features. The dataset, provided in CSV format, was uploaded to the Anaconda, where visualizations such as heatmaps and scatter plots were generated. These graphs helped to identify feature correlations and understand feature importance. The resulting visualizations were saved in TIF format for further analysis. Additionally, EDA was performed using Python to better understand data distributions and relationships, ensuring the selected features were relevant for model development [20].

#### Data Visualization with PCA

PIMA datasets were subjected to unsupervised ML technique PCA using trail version XLSTAT software version 2014.5.03. PCA and Biplot were obtained for the correlation and clustering of the dataset.

### 2.3. Pre-Processing

#### 2.3.1. Normalization

The collected PIMA dataset was pre-processed to find out missing values, and scaling of the dataset was performed using the min–max method [20,21] using Equation (1):

(1) $$Normalized\;z=\frac{z-min(z)}{\max\left(z\right)-min(z)}$$

The dataset used in this study was already clean, with no missing values. It contained 768 records and included eight attributes related to the participants who were enrolled and completed the study (Table S1).

#### 2.3.2. Feature Extraction

The numerical features (e.g., glucose, BMI) were normalized to ensure all attributes were on a similar scale. Features were normalized or standardized to improve ML model performance. PCA was performed to reduce dimensionality and extract important components to avoid multicollinearity and improve computational efficiency.

To improve the performance of ML models, the numerical features (e.g., glucose, BMI) were normalized to ensure all attributes were on a similar scale. Normalization or standardization of features helps optimize model accuracy and performance. Principal Component Analysis (PCA) was performed to reduce the dataset’s dimensionality by extracting the most important components. This process not only improved computational efficiency but also minimized the risk of multicollinearity among features [22].

#### 2.3.3. Dataset Splitting

After data pre-processing, the Pima dataset was divided into training and testing subsets to evaluate ML model performance. The dataset was split into a Training Set (70%) that was used to train the ML models. The Testing Set (30%) was used for model evaluation on unseen data [23]. Proper dataset splitting ensures that the model generalizes well to new, unseen data. Different ML models were generated, and the results were compared using different parameters such as TPR, TNR, accuracy, recall sensitivity, precision, specificity, and F1-score.

### 2.4. ML Models Development

After the dataset splitting, various ML models were trained on the training dataset. RF, DT, NB, and LR ML models were developed by using the Pima dataset. The models were trained on the 70% training dataset. Python libraries such as open-source scikit-learn (version 0.18) ML toolkit were used for implementing these models. Python package was used for the construction, modification, and validation of each model [24].

### 2.5. Model Performance Evaluation

After developing the ML models, their performance was evaluated using the 30% testing dataset. The models were assessed and compared based on six evaluation metrics, including Precision, Sensitivity (Recall), Specificity, F-Score, Error Rates, and Accuracy. To measure these metrics, Python code was used to generate confusion matrices, and ROC curves. The confusion matrix (Table 2) displayed the predicted vs. actual outcome classes for the testing set, from which the above metrics were calculated. These tools provided a detailed analysis of each model’s performance and its ability to accurately classify diabetes cases. In addition, five-fold cross-validation was applied to the training and testing datasets to further assess the model performance. This technique ensures that the models are evaluated on different subsets of the data, helping to avoid overfitting and improving the model’s generalization. Finally, the performance of the tuned models was evaluated on the testing dataset, and their results were compared based on the metrics of Accuracy and others.

Where TP = true positive, TN = true negative, FP = false positive, and FN = false negative.

- True Positive (TP): Correctly predicted positive cases.
- False Positive (FP): Incorrectly predicted positive cases (actually negative).
- True Negative (TN): Correctly predicted negative cases.
- False Negative (FN): Incorrectly predicted negative cases (actually positive).

Below are the formulas for the evaluation metrics used to assess the performance of different models.

The Accuracy of a prediction refers to the percentage of all samples that have been predicted correctly. It is calculated by dividing the sum of true positives and true negatives by the total number of predictions made. It was calculated using the following formula, Equation (2).

(2) $$Accuracy=\frac{\left(TP+TN\right)}{\left(TP+TN+FP+FN\right)}$$

Precision is represented as a ratio of true positive results to the sum of true-positive and false-positive results. Equation (3) was used to calculate precision.

(3) $$Precision=\frac{TP}{\left(TP+FP\right)}$$

Sensitivity, also called Recall or True-Positive Rate, measures the proportion of actual positives that are correctly identified. It was calculated using Equation (4):

(4) $$TPR\left(recall\right)=\frac{TP}{\left(FN+TP\right)}$$

Specificity measures the proportion of actual negatives that are correctly identified. It is also known as the True-Negative Rate. To calculate specificity Equation (5) was used.

(5) $$TNR=\frac{TN}{\left(FP+TN\right)}$$

The Error rate represents the proportion of all instances that were incorrectly classified. It is the complement of accuracy and gives an overall measure of how often the model makes mistakes in its predictions. It was calculated using the following formula as given in Equation (6):

(6) $$Error\;rate=\frac{FP+FN}{TP+TN+FP+FN}$$

The F1-score is the harmonic mean of precision and recall (sensitivity). It is a measure of a test’s accuracy and is used when there is an uneven class distribution. The F1-score balances precision and recall, providing a single metric to evaluate a model’s performance. Specifically, it is calculated by dividing the product of the precision and sensitivity by the sum of the precision and sensitivity and multiplying the result by two [25]. It is calculated as following Equation (7):

(7) $$F-Measure=\frac{2TP}{\left(2TP+FP+FN\right)}$$

## 3. Results

### 3.1. Dataset Characteristics

The risk of Type 2 diabetes has significantly increased globally across all age groups, particularly among individuals who are obese, have an unhealthy lifestyle, or have a family history of the condition. In this study, we focused on analyzing eight different attributes as independent variables to predict the presence or absence of diabetes. The first step in the analysis was to examine the correlation between various features. It was observed that skin thickness tends to increase with BMI [26]. Additionally, glycosylated hemoglobin (HbA1c) provides important information about a patient’s average blood glucose level over the past three months [27].

Previous research has also shown that pregnant women are more likely to develop a condition known as gestational diabetes, which is a serious complication for both the mother and child, as this is a significant risk factor, it was included as a key variable in our study [28].

The mean for various features was also calculated and it was found that the mean glucose level was around 72 mg/mL. The blood pressure distribution graph depicted that the mean blood pressure was around 80 mmHg, skin thickness had a mean of 26.60, the mean BMI was 32.4, for the diabetic pedigree function it was 0.47, and the average age was 33.24 years Table 3.

The selected attributes have important significance in the prediction of diabetes insulin level decreases with the development of IDDM, but in NIDDM, it is not always that the level of insulin decreases; in this condition, body cells are not able to absorb glucose from the blood into the cell because of cellular dysfunction.

**Figure 3:**
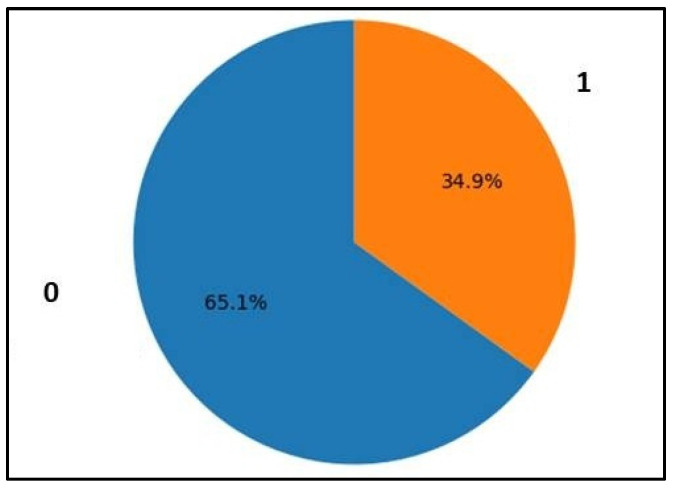
The outcome of PIMA dataset 0 denotes non-diabetic and 1 represents diabetic subject.

Figure 3 shows a pie chart representing the distribution of the outcome variable of the PIMA dataset. The dataset is used to predict the presence or absence of diabetes. The outcome variable, which indicates whether the patient has diabetes (1) or not (0), is displayed on the pie chart with percentage contribution. The results show that 500 non-diabetic subjects, which accounts for 65.1%, and 268 diabetic subjects, accounting for 34.9% of the total subjects, were enrolled in this study.

Outcomes were represented in zero and one: zero represents non-diabetic subjects, and there were 500 instances where the subjects did not have diabetes; one denotes diabetes subjects, and there were 268 instances where the subjects had diabetes.

The plot was generated using Python, specifically with the Seaborn or Matplotlib library to visualize the count of each class in the Outcome column of the dataset.

The objective of the PIMA study was to predict diabetes among pregnant ladies, as developing diabetes is more likely among pregnant ladies and may also lead to a post-gestational period as at this age prediction plays a very important role. If we can detect diabetes early in life, we can delay its development at a later age using proper diet and lifestyle modifications; in addition, complications associated with it can be reduced in this crucial period of mother and child [29]. The size of the dataset of 768 ladies was moderate for the development of a robust model, but the enrolment of ladies in clinical studies is limited; for the same reason, the study is very important to predict diabetes and extrapolate the results to other female populations.

### 3.2. Principal Component Analysis

PCA is an unsupervised statistical method used to reduce the dimensionality of a dataset. The number of feature combinations that can be obtained grows exponentially with the number of dimensions, making it more computationally demanding to collect a representative sample of the data and more costly to carry out tasks like clustering or classification. Applying ML techniques to high-dimensional data suffers from problems such as overfitting, larger computation time, and decreased ML model accuracy [30]. These issues are known as the “curse of dimensionality”.

PCA uses orthogonal transformations to convert a set of features into a smaller subset of features that are not linearly correlated to each other. There is not much information loss in this process. The principal components that we obtain are sorted based on the variance of the dataset with the first component (PCA1) covering the maximum variance, and each subsequent component covering a lesser value of variance [31].

Suppose {**x**(*t*)}; *t* = 1, …, *n* represents a random dataset having several features and instances having a mean equal to zero.

The covariance matrix of **x**(*t*) will be

(8) $$Cov\left(x\right)=\frac{\sum_{t=1}^{n}\left[x\left(t\right){x(t)}^{T}\right]}{\left(n-1\right)}$$

In Principal Component Analysis the linear transformation from **x**(*t*) to **y**(*t*) can be calculated as in Equation (9)

(9) $$y\left(t\right)=M^{T}x(t)$$

where *M* is an *n* × *n* orthogonal matrix. The *i*th column, of the sample covariance matrix Cov(x), is equivalent to the *i*th eigen-vector.

**Figure 4:**
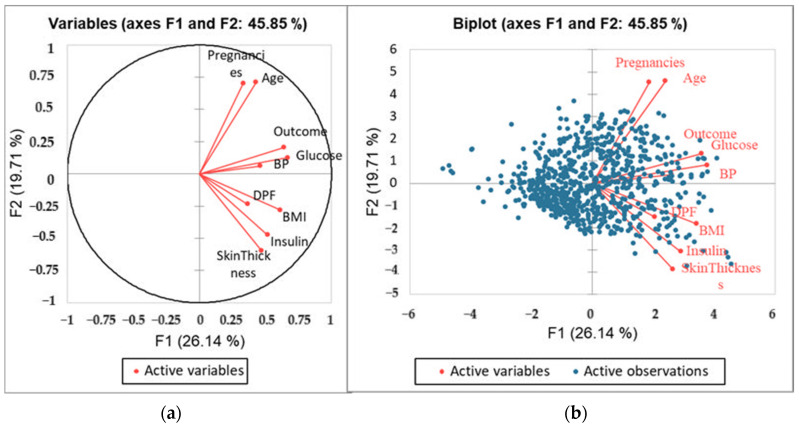
(**a**) PCA Showing active variables, (**b**) biplot analysis of the dataset showing active variables and active observations.

The PCA plot displays the relationships among variables (features) and their contribution to the first two principal components (F1 and F2). The closer a variable is to the circle’s edge, the stronger its correlation with the principal components. Variables that are close to each other showed positively correlated, while those on opposite sides are negatively correlated. The first two principal components account for 45.85% of the total variance (F1: 26.14%, F2: 19.71%) (Figure 4).

Glucose, BMI, and Insulin contribute significantly to the first component (F1). Age and Pregnancy contribute to both F1 and F2. Skin thickness and DPF are less strongly correlated with these components. Strong positive correlations between some variables, such as BMI and Glucose, are evident from their similar arrow directions.

The biplot (Figure 4b) combines the variable loadings (red arrows) with the projection of observations (blue points) in the principal component space. The blue points represent individual observations projected into the PCA space. Observations near a variable’s arrow are influenced by that variable. Points close to Glucose or BMI arrows suggest that these observations have high values for those variables. Points distributed near the origin are less influenced by any single variable.

Eigenvalues are scalars associated with a square matrix that provide insights into its properties, particularly in linear transformations. They are used in many mathematical, statistical, and ML applications, such as Principal Component Analysis (PCA).

For a square matrix A, an eigenvalue λ, lambda λ satisfies the following equation

(10) Av=λv

where

A is the square matrix;

v is a non-zero vector called the eigenvector;

λ is the eigenvector.

We also calculated eigenvalues for all factors, where it was found that factor F1 accounts for 26.139%, F2 19.71 (Table 4), which cumulatively accounts for 45.85% of the variability in the dataset.

Whereas other factors account only for the 55.14% variation in the dataset.

The squared cosines of variables, often used in factor analysis or principal component analysis (PCA), represent the proportion of a variable’s variance that is explained by each factor or principal component. It provides insight into how well the variable aligns with the respective factor/component.

The squared cosines table for the PIMA dataset was generated using the PCA analysis. The values in bold correspond for each variable to the factor for which the squared cosine is the largest; glucose and BMI have the largest contribution for F1, and pregnancies, skin thickness, and age were the important factors for F2. Similarly, F3 has blood pressure, F4 diabetic pedigree function, and F5 insulin (Table 5).

### 3.3. Scatter Pot

A scatter plot is a type of graph that uses dots to represent data points. The position of each dot on the horizontal and vertical axes corresponds to the values of two different variables. Scatter plots are used to identify relationships between variables.

**Figure 5:**
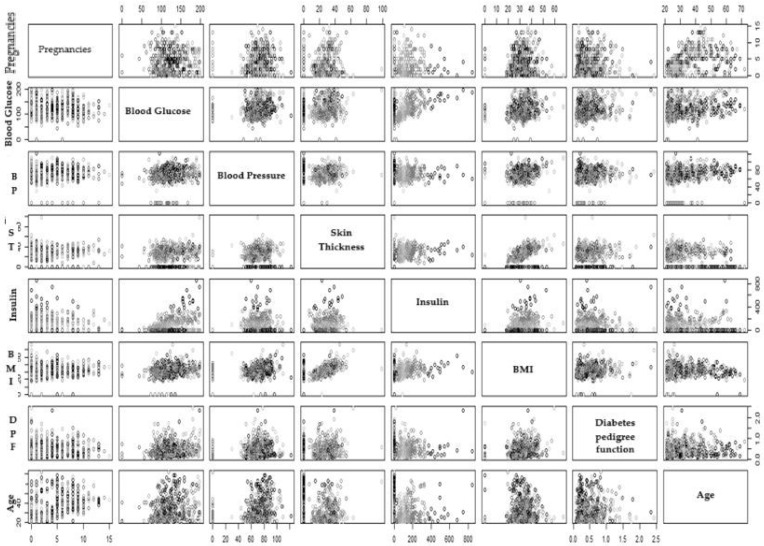
Scatter plot for different attributes of the PIMA dataset.

If there is a positive correlation between two variables, the dots on the scatter plot trend upward from left to right. If there is a negative correlation, the dots trend downward from left to right. And if there is no correlation, the dots randomly scatter, see Figure 5.

In our dataset, we found a strong positive correlation between BMI and skin thickness, insulin, and blood glucose, while age does not have a positive correlation with the number of pregnancies, as can be seen in Figure 5. Other factors do not have a positive or negative correlation with each other, dots are scattered throughout the scattered plot. These relationships also verify the results obtained in [32]; similar results were obtained in heatmap analysis in our study.

### 3.4. Correlation Analysis

**Figure 6:**
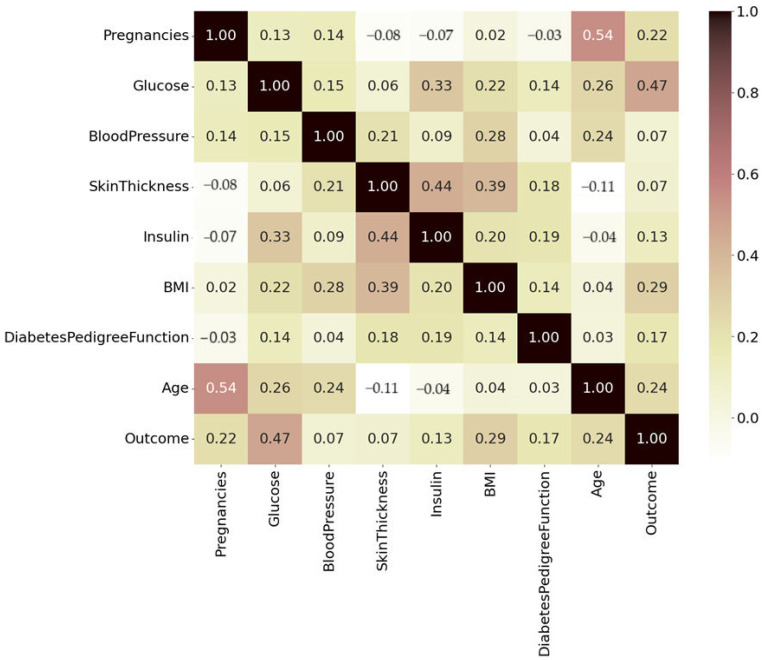
Heatmap analysis of the PIMA dataset.

Correlation Analysis is used to measure the strength of the relationship between different features. Correlation analysis was carried out to find the association among different attributes of the dataset. A heatmap was generated to visualize correlations. The heatmap graph shows a correlation in color format and value between a 0 to 1.0 positive and 0 to −1 negative correlation see Figure 6.

The colors in this image are primarily shades of yellow, brown, and white with some hints of red and pink. The color intensity varies based on the correlation values:

- Darker shades, dark brown to black, represent strong positive correlations of 0.8 to 1.0.
- Pink and red (0.4 to 0.79) are moderately positive, and lighter shades of pale yellow and white indicate weaker or near-zero correlations (0.2 to 0.39).
- Negative correlations are faint brown.

In our study, age with skin thickness and insulin have a negative correlation. This could be because insulin secretion reduces as age increases due to a decline in β cells of pancreatic islets [33]; similarly, skin thickness also decreases in the elderly population [34], although the mean age of the subjects was 33, so it shows a weak-to-moderate negative correlation in our study. We also found that the number of pregnancies has a weak negative correlation, and it was evident from the study [35] that insulin resistance increases in pregnant ladies because of their hormonal changes. The DPF is a feature commonly used in diabetes prediction datasets (like the famous Pima Indians Diabetes Dataset). It quantifies a person’s genetic predisposition to diabetes based on family history [32]. The diabetic pedigree function does not show a strong positive or negative correlation with other attributes. However, BMI, insulin, and blood glucose levels exhibit a slight positive correlation with the diabetic pedigree function. The BMI attribute does not show any correlation with age or the number of pregnancies. However, it has a moderate correlation with blood pressure, skin thickness, and blood glucose levels. The age of the subject has a moderate positive correlation with the number of pregnancies. This is understandable, as older individuals are more likely to have had more pregnancies over time.

### 3.5. ML Models

#### 3.5.1. Decision Tree

A DT is a type of tree structure that resembles a flowchart, with core nodes representing features, branches representing rules, and leaf nodes representing the algorithm’s outcome. It is a flexible supervised machine learning approach that may be applied to regression and classification issues alike. It is among the most potent algorithms. Additionally, RF uses it to train on various subsets of training data, making it one of the most potent ML algorithms.

DT broadly classified the dataset into two classes based on the blood sugar level. A total of 62% of subjects have blood sugar levels less than 128 mg/mL, among them 42% are below the age of 32 years, and they are also the majority of the dataset, as shown in Figure S1. On the other hand, 32% of the subjects have a blood sugar level of more than 128 mg/mL, among them 27% of the subjects have a BMI above 30, and 10% of the subjects have a BMI less than 30. The confusion matrix for DT is shown in Table 6.

This means the model correctly predicted 119 diabetic cases as diabetic and 47 non-diabetic cases as non-diabetic, but it also misclassified 37 non-diabetic cases as diabetic and 28 diabetic cases as non-diabetic.

#### 3.5.2. Random Forest

An RF is a powerful ML algorithm that utilizes the combined power of multiple DTs to make predictions. Its functions are analogous to a DT, but it creates random combinations of characteristics for classification purposes rather than splitting at a single attribute. More analysis can be undertaken as a result, which increases this model’s accuracy in comparison to a single tree model. The confusion matrix of the developed RF model is shown in Table 7.

This means the model correctly predicted 129 diabetic cases as diabetic and 55 non-diabetic cases as non-diabetic, but it also misclassified 29 non-diabetic cases as diabetic and 18 diabetic cases as non-diabetic.

#### 3.5.3. Naïve Bayes

The “naive” presumption that each of the predictor variables is unaffected by one another and, hence, will not affect each other makes NB an oversimplified model. To calculate the probability of the class result and generate a prediction, it applies Bayes probabilistic concepts to all attributes. The developed confusion matrix for NB is given in following Table 8.

This means the model correctly predicted 127 diabetic cases as diabetic and 51 non-diabetic cases as non-diabetic, but it also misclassified 33 non-diabetic cases as diabetic and 20 diabetic cases as non-diabetic.

#### 3.5.4. Logistic Regression

A statistical technique called LR makes use of mathematics to determine the correlations between two data components. The value of one of those parameters is then predicted depending on the other using this relationship. In our dataset, eight different independent variables were used to predict diabetes, and the developed RF model’s confusion matrix is given in Table 9.

This means that the model correctly predicted 114 diabetic cases as diabetic and 49 non-diabetic cases as non-diabetic, but it also misclassified 35 non-diabetic cases as diabetic and 33 diabetic cases as non-diabetic.

The results from all the developed models for predicting diabetes are shown in Table 8. The findings indicate that RF achieved the highest accuracy of 80%, as compared to NB of 77%, DT 72%, and LR had the lowest accuracy of 70.5%. Additionally, RF had the lowest error rate of 20% as compared to other developed models; similarly, RF also demonstrated better performance compared to the other models Table 10.

**Figure 7:**
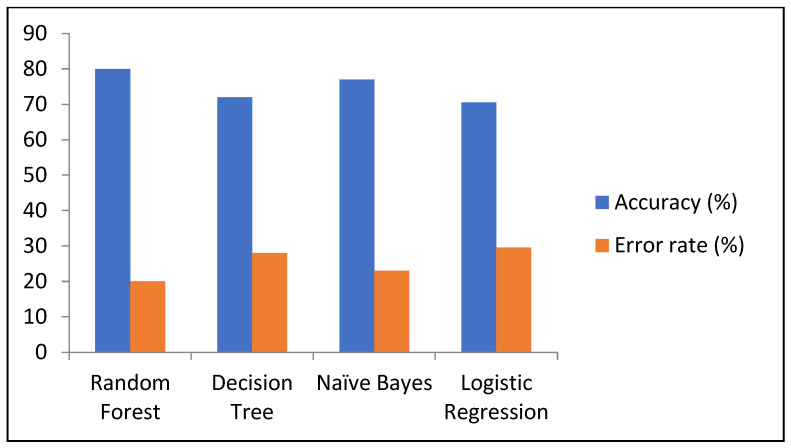
Comparison of performance in terms of accuracy and error rate.

Based on these results, RF outperforms other models in most evaluation metrics, making it the most effective model for predicting diabetes in this dataset. The comparison of accuracy and error rates of different ML models is shown the Figure 7.

**Figure 8:**
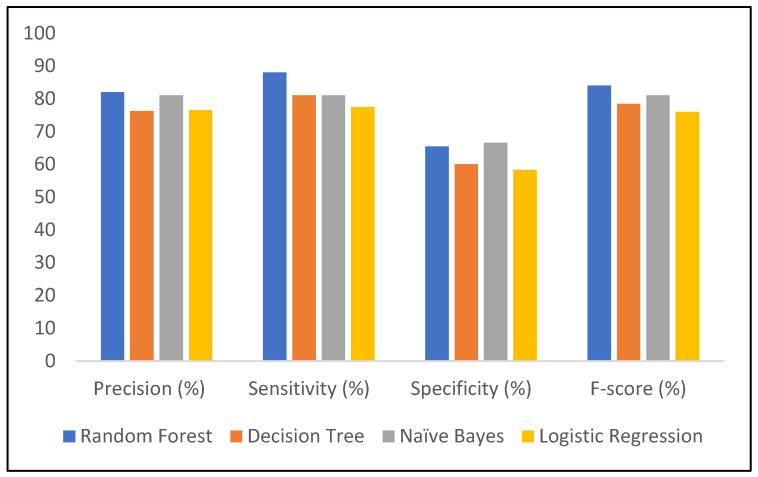
Precision. Sensitivity, specificity, and F-score of developed ML models.

A comparison of the entire developed model in terms of Precision, Sensitivity, specificity, and F-score is shown in Figure 8. The RF model has the best precision as compared to other models; DT and LR have almost similar precision. Overall, by looking at all the parameters: sensitivity, specificity, and F-score, it can be seen that RF gives the best results.

In comparing all the developed models, RF offers the highest accuracy of 80% among all models, and it is the most effective at identifying positive cases with a sensitivity of 88%, with good precision of 82%, and F-score of 84%, indicating a balanced performance between precision and recall. The model struggles more with identifying true negatives compared to others, with a low specificity of 65.4%. Overall, RF performs best particularly for tasks where sensitivity and overall accuracy are priorities.

### 3.6. ROC Curve

**Figure 9:**
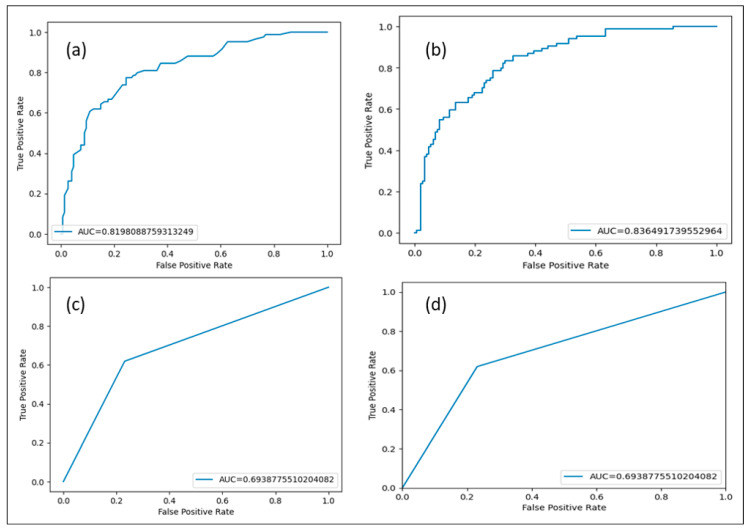
ROC curve for the developed ML: (**a**) Naïve Bayes AUC 0.81, (**b**) Random forest AUC 0.83, (**c**) Decision Tree achieved AUC 0.69, and (**d**) Logistic regression achieved AUC 0.69.

The Receiver Operating Characteristic (ROC) curve is a graphical representation used to evaluate the performance of a binary classification model. It shows the trade-off between the TPR and the FPR at various thresholds. We have developed an ROC curve for our study, and the results are presented in Figure 9.

A value of ROC represents a perfectly developed model, an AUC value below 0.5 indicates a poor model, an AUC between 0.7 to 0.8 suggests the developed model may be acceptable, 0.8 to 0.9 AUC represents the good model, and above 0.9 represents an excellent model [36,37]. In our study, the RF model ROC shows better performance, which we also observed in the performance analysis of the developed model, as depicted in Table 8. The AUC for the RF was 0.83 as compared to NB 81, followed by DT and LR 0.69,

### 3.7. Cross Validation

In ML, cross-validation is a technique used to evaluate the performance of a model on unseen data. It is particularly useful for preventing a common problem called over-fitting. We used five-fold cross-validation on the training and testing dataset, and we also used five-fold cross-validation in our experiment as it offers the following advantages.

- It achieves a balance between computational cost and performance evaluation reliability.
- It provides sufficient training data for each fold, especially for moderately large datasets.
- It is a common practice supported by the literature, particularly in experiments involving iterative tuning or resource constraints.
- Five-fold cross-validation methods are used for small-to-large datasets; in other diabetic research, five-fold CV offers good results [38]. The results obtained in our study are shown in Table 11, and it can be seen that NB has a slightly better average accuracy of 0.76 on the testing dataset as compared to RF 0.75, which is statistically not significant.

The results show that LR has better average accuracy on the training dataset as compared to other developed models, and DT has the least accuracy on both the training and testing datasets.

## 4. Conclusions

Diabetes is a metabolic disorder characterized by high blood sugar levels, which can lead to various life-threatening complications and reduce life expectancy. Early detection is crucial to preventing or delaying the development of these complications. In this study, we utilized the PIMA dataset; the collected dataset was analyzed using PCA and heatmap analytical tools, and ML-based prediction models were developed using this dataset, and performance of the developed model was evaluated on training and testing datasets using Python. The developed models were evaluated using different evaluation techniques such as ROC, accuracy, precision, error rate, etc. The results showed that RF outperformed the other models, achieving the highest accuracy, sensitivity, and precision, with the lowest error rate for predicting diabetes in the PIMA dataset.

## 5. Way Forward

In our dataset, only female patients aged 21 and above were enrolled with the mean age of the subjects being 31. This means that the population under this research was quite young and had a lower chance of developing early signs and symptoms of this disease. In our future research work, we would like to use a dataset with a mean age of 50 years consisting of both males and females to explore the prediction of diabetes in large populations and broader coverage with the inclusion of more numbers of attributes such as type of food intake, average time spent in exercise, does the subject perform yoga daily, and many more.

## Figures and Tables

**Table 1:** Datasets information.

Attributes	Inference
Pregnancies	Number of times the person has been pregnant
Blood Glucose	Blood glucose level on testing
Blood Pressure	Diastolic blood pressure
Skin Thickness	Skin fold thickness of the triceps
Insulin	Amount of insulin in a 2 h serum test
BMI	Body mass index
DPF	DPF calculates a score that represents the likelihood of diabetes influenced by hereditary factors.
Age	Age of the person
Outcome	The person is predicted to have diabetes or not

**Table 2:** Confusion matrix template.

	Predicted Positive	Predicted Negative
Actual positive	True positive	False negative
Actual negative	False positive	True negative

**Table 3:** PIMA Indian dataset statistics.

	Pregnancies	Glucose	Blood Pressure	Skin Thickness	Insulin	BMI	DPF	Age
Count	768	768	768	768	768	768	768	768
Mean	3.84	121.60	72.20	26.60	118.68	32.4	0.47	33.24
std	3.36	30.40	12.10	9.60	93.08	6.80	0.33	11.76
Min	0.00	44.00	24.00	7.00	14.00	18.20	0.07	21.00
25%	1.00	99.70	64.00	20.50	79.70	27.50	0.24	24.00
50%	3.00	117.00	72.00	23.00	79.70	32.0	0.37	29.00
75%	6.00	140.25	80.00	32.00	127.25	36.60	0.62	41.00
max	17.00	199.00	122.00	99.00	846.00	67.10	2.42	81.00

**Table 4:** The eigenvalue for the PCA generated using the PIMA dataset.

	F1	F2	F3	F4	F5	F6	F7	F8	F9
Eigenvalue	2.353	1.774	1.120	0.882	0.845	0.735	0.488	0.418	0.385
Variability (%)	26.139	19.715	12.447	9.799	9.385	8.165	5.427	4.646	4.277
Cumulative %	26.139	45.853	58.300	68.100	77.485	85.650	91.077	95.723	100.000

**Table 5:** Squared cosines of the variables.

	F1	F2	F3	F4	F5
Pregnancies	0.110	**0.494**	0.030	0.023	0.038
Glucose	**0.449**	0.016	0.172	0.094	0.010
Blood Pressure	0.212	0.004	**0.444**	0.000	0.003
Skin Thickness	0.222	**0.357**	0.097	0.005	0.025
Insulin	0.266	0.224	0.022	0.008	**0.357**
BMI	**0.371**	0.078	0.071	0.023	0.235
DPF	0.133	0.054	0.091	**0.673**	0.021
Age	0.183	**0.504**	0.018	0.026	0.035
Outcome	**0.406**	0.043	0.174	0.029	0.121

Values in bold correspond for each variable to the factor for which the squared cosine is the largest.

**Table 6:** Confusion matrix DT.

	Predicted Positive	Predicted Negative
Actual positive	119	28
Actual negative	37	47

**Table 7:** Confusion matrix for the developed Random Forest.

	Negative Positive	Predicted Negative
Actual positive	129	18
Actual negative	29	55

**Table 8:** Confusion matrix for the Naïve Bayes developed model.

	Predicted Positive	Predicted Negative
Actual positive	127	20
Actual negative	33	51

**Table 9:** Confusion matrix for logistic regression.

	Predicted Positive	Predicted Negative
Actual positive	114	33
Actual negative	35	49

**Table 10:** Performance analysis of the developed ML algorithms.

Model	Accuracy (%)	Precision (%)	Sensitivity (%)	Specificity (%)	F-Score (%)	Error Rate (%)
RF	80	82	88	65.4	84	20
DT	72	76.2	81	60	78.4	28
NB	77	81	81	66.6	81	23
LR	70.5	76.5	77.5	58.3	76	29.5

**Table 11:** Cross-validation on training and testing datasets.

Algorithms	Training Dataset						Testing Dataset					
	K-1	K-2	K-3	K-4	K-5	Avg	K-1	K-2	K-3	K-4	K-5	Avg
RF	0.75	0.70	0.78	0.71	0.75	0.74	0.72	0.82	0.71	0.76	0.71	0.75
DT	0.68	0.72	0.71	0.71	0.72	0.71	0.59	0.78	0.69	0.78	0.71	0.71
NB	0.78	0.69	0.76	0.72	0.73	0.74	0.78	0.78	0.73	0.73	0.76	0.76
LR	0.84	0.72	0.78	0.72	0.76	0.76	0.76	0.76	0.67	0.69	0.73	0.72

## Data Availability

The original data associated with the study are available with the manuscript; for further data, readers may inquire to the corresponding author.

## Supplementary Materials

The following supporting information can be downloaded at: https://www.mdpi.com/article/10.3390/healthcare13010037/s1, Table S1: PIMA dataset Figure S1: Decision tree developed using PIMA dataset.
